# The concentration of lidocaine and mepivacaine measured in synovial fluid of different joints of horses after single intra-articular injection

**DOI:** 10.3389/fvets.2022.1007399

**Published:** 2022-11-10

**Authors:** Ditte M. T. Adler, Elin Jørgensen, Claus Cornett

**Affiliations:** ^1^Department of Veterinary Clinical Sciences, Faculty of Health and Medical Sciences, University of Copenhagen, Taastrup, Denmark; ^2^Department of Pharmacy, Faculty of Health and Medical Sciences, University of Copenhagen, Copenhagen, Denmark

**Keywords:** lameness diagnosis, local anesthetics, equine lameness, lidocaine, mepivacaine

## Abstract

**Objective:**

To determine the synovial fluid (SF) concentrations of lidocaine and mepivacaine after intra-articular injection with clinically relevant doses to the distal interphalangeal (DIP), metacarpophalangeal (MCP), middle carpal (MC), and tarsocrural (TC) joint at two different time points after injection in order to be able to compare concentrations with previously established concentrations associated with cytotoxicity and antimicrobial activity.

**Procedures:**

In the first of two experiments, 20 joints (5 MC, 5 MCP, 10 DIP joints) of five horses under general anesthesia were injected with clinically referenced doses of 2% lidocaine. Simultaneously, the horses had 19 joints (5 MC, 5 MCP, 9 DIP joints) injected with clinically referenced doses of 2% mepivacaine. Synovial fluid samples were collected ~7 min after injection. In experiment 2, 23 joints of seven horses under standing sedation were injected with clinically referenced doses of 2% lidocaine. Similarly, the horses had 21 joints injected with 2% mepivacaine. Synovial fluid samples were collected ~23 min after injection. The concentration of mepivacaine and lidocaine in the obtained SF samples was assessed using high-performance-liquid-chromatography with mass spectrometry detection (HPLC MS).

**Results:**

Synovial fluid was obtained 6.8 ± 1.5 (experiment 1) and 23 ± 4.3 (experiment 2) min following intra-articular injection of mepivacaine and lidocaine. Synovial fluid concentrations of experiment 1 for lidocaine and mepivaciane were 6.46–19.62 mg/mL (mean 11.96 ± SD 3.89 mg/mL) and 5.01–13.38 mg/mL (mean 8.18 ± SD 1.76 mg/mL), respectively. In experiment 2, concentrations were 2.94–10.40 mg/mL (mean 6.31± SD 2.23 mg/mL) for lidocaine and 2.10–8.70 mg/mL (mean 4.97 ± SD 1.77 mg/mL) for mepivacaine.

**Conclusions and clinical relevance:**

Intra-articular LA injections in horses resulted in SF concentrations above those previously associated with cytotoxic effects *in vitro* but also above those associated with beneficial antimicrobial activities. Local anesthetic concentration was 33–60% lower after 23 min (experiment 2) than after 7 min (experiment 1).

## Introduction

Intra-articular local analgesia with LAs (local anesthetics) as part of lameness investigations in horses is a fundamental and invaluable diagnostic tool. The LAs used most commonly for this purpose are 2% mepivacaine and 2% lidocaine (1, 2). Local anesthetics as we know them today were first discovered and prepared (lidocaine) in 1944 (3). As early as 1963, their use for abolishing lameness in the horse was described (4), but it was not until the past decades, that LAs were associated with detrimental effects on joints (5–8). Specifically, concentrations of 1–2% lidocaine and mepivacaine possess chondrotoxic and synoviotoxic effects *in vitro* (7–11). *In vivo*, single intra-articular injections of 2% mepivacaine and lidocaine as used during lameness diagnosis resulted in a catabolic cartilage response as well as in joint inflammation in horses (12).

Local anesthetics have not only been associated with harmful side effects in the joint, but have also been linked to a beneficial side effect, namely an inherent antimicrobial activity (13–15). This activity has been verified for both lidocaine and mepivacaine against common equine isolates at concentrations ranging from 1.3 to >15 mg/mL (16).

While LA injection doses to some degree are based on studies (17, 18), the majority of intra-articular doses for abolishing lameness in the horse are based on expert clinical recommendation (1). Albeit, the minimum synovial fluid concentration of LAs to abolish lameness is unknown, it has been estimated that mepivacaine concentrations of >0.3 mg/mL are sufficient to abolish equine joint related lameness (23).

The actual concentration in the synovial fluid (SF) of a joint after injection of recommended doses remains unexplored. We therefore aimed at determining LA SF concentrations after intra-articular injection at two different time points to a number of different equine joints. This in order to explore if obtained *in vivo* LA concentrations exceeds those concentrations previously established to be cytotoxic to articular cells *in vitro* and above those concentrations previously associated with beneficial antimicrobial activities.

## Materials and methods

### Animals

All study procedures were approved by the ethical committee of the University of Copenhagen Large Animal Teaching Hospital and the Danish Animal Experiments Inspectorate (license No. 2015-15-0201-00608). The Danish Animal Experiments Inspectorate works under the European Directive 2010/63/EU. All procedures were conducted in accordance with the Danish Animal Testing Act, and care of the horses was in accordance with institutional guidelines. All horses were owned by and maintained at the University of Copenhagen for teaching and research purposes.

The study consisted of two experiments in order to collect SF at two different time points after LA injection. We aimed at collecting SF from injected joints 5 and 20 min after LA injection of horses in experiment 1 and 2, respectively. Experiment 1 involved five adult mares with a mean ± SD age of 5.9 ± 1.8 years, body weight of 470 ± 41 kg. Experiment 2 involved seven adult horses (six geldings, one mare) with a mean ± SD age of 7.1 ± 2.5 years, body weight of 498 ±37 kg. The horses in both experiments were determined to be healthy, without clinical evidence of disease in the DIP, MCP, MC, and TC joints and free of lameness based on physical and subjective lameness examinations performed immediately prior to each experiment. Horses of experiment 1 were enrolled in a different terminal study on atrial fibrillation performed under general anesthesia (license No. 2016-15-0201-01128). Horses of experiment 1 had been in persistent experimentally induced atrial fibrillation for 43.3±4.7 days prior to induction of anesthesia. Apart from that, the pre-anesthetic physical examination, hematologic and blood biochemical analyses revealed no abnormalities (data not shown). Experiment 2 was carried out in standing sedation immediately prior to euthanasia for reasons unrelated to this study.

At our institution, we constantly aim at maximizing use of each individual experimental animal in order to follow 3Rs principle. Therefore, horses of experiment 1 was also enrolled in a different study as described above, which included general anesthesia. Inclusion of these horses gave us the opportunity to retrieve SF quickly after intra-articular LA-injection, which was a specific research interest of our study.

### Experiment 1

In experiment 1, horses were subjected to intra-articular injection of 2% lidocaine (=20 mg/mL) and 2% mepivacaine (=20 mg/mL) while under general anesthesia. The left middle carpal (MC) (*n* = 5) and metacarpophalangeal (MCP) joint (*n* = 5) were injected with 10 mL mepivacaine. The left distal interphalangeal (DIP) joint of the forelimb was injected with 6 ml (*n* = 1) or 10 mL (*n* = 3) mepivacaine. The left DIP joint of the hind limb was injected with 5 mL (*n* = 4) or 10 mL (*n* = 1) mepivacaine. The right MC (*n* = 5) and MCP joint (*n* = 5) were injected with 10 mL lidocaine. The right DIP joint of the forelimb was injected with 6 mL (*n* = 1) or 10 mL (*n* = 4) lidocaine. The right DIP joint of the hind limb was injected with 5 mL (*n* = 4) or 10 mL (*n* = 1) lidocaine.

### Experiment 2

In experiment 2, horses were subjected to intra-articular injection of 2% lidocaine and 2% mepivacaine while under standing sedation. The left MC (*n* = 7) and MCP joint (*n* = 4) were injected with 10 mL mepivacaine. The left DIP joint of the forelimb was injected with 5 mL (*n* = 1) or 10 mL (*n* = 3) of mepivacaine. The left tarsocrural (TC) joint was injected with 20 (*n* = 1) or 30 (*n* = 5) mL mepivacaine. The right MC (*n* = 7), MCP joint (*n* = 6) and DIP joint of the forelimb (*n* = 4) was injected with 10 mL lidocaine. The right TC joint was injected with 20 (*n* = 1) or 30 mL (*n* = 5) of lidocaine.

#### The use of different volumes for the distal interphalangeal joint and the tarsocrural joint

For several joints, recommendations for achieving intra-articular anesthesia are provided as intervals. Specifically, for the DIP joint and TC joint 5–10 and 20–30 mL of 2% lidocaine/mepivacaine are recommended, respectively (1), and some studies recommend use of 6 ml for the DIP joint (17). As we wanted to mimic clinical settings we included different volumes for the DIP joint (5, 6, and 10 mL) and TC joint (20 and 30 mL). Furthermore, as no information on exact joint size and volume exists between front and hind limbs, differences may exist. For complete transparency of our results we therefore list measured concentrations based on whether front or hind limb was used for the specific volume.

### Experimental procedures

In experiment 1, horses were premedicated through an indwelling jugular vein catheter with 0.01 mg/kg detomidine hydrochloride and 0.03 mg/kg acepromazine followed by 0.01 mg/kg butorphanol intravenously after 5 min. Anesthesia was induced with 0.75 mg/kg zolazepam and 0.75 mg/kg tiletamine and maintained with isoflurane in 100% oxygen using intermittent positive pressure ventilation. Horses were in right lateral recumbency and joints were clipped and aseptically prepared for arthrocentesis. Arthrocenteses were performed with size 20 gauge needles. After intra-articular injection of horses in experiment 1, legs with injected joints were manually fully flexed and extended 30 times to allow for distribution of drug within the joints. Hereafter SF was obtained from the injected joints in the same order they were injected approximately 7 min after administration. Time from injection to retrieval of SF was recorded for each individual joint.

In experiment 2, horses were sedated intravenously through an indwelling jugular vein catheter with 0.01 mg/kg detomidine hydrochloride, 0.03 mg/kg acepromazine and 0.01 mg/kg butorphanol. Joints were clipped and aseptically prepared for arthrocentesis. Arthrocentesis of all joints were performed with 20 gauge needles. Joints were excluded from the study if horses were uncooperative during injection or if not all LA were deposited intra-articularly (i.e., if needle and syringe disengaged during injection and part of the LA escaped from the syringe without entering the joint). After intra-articular injection, horses were walked for 5 min to allow for distribution of drug within the joint before being subjected to euthanasia by intravenous injection with 160 mg/kg pentobarbital. Synovial fluid samples were obtained immediately after euthanasia from all injected joints in the same order they were injected approximately 23 min after injection. Time from injection to aspiration of SF was recorded for each individual joint.

Synovial fluid samples from experiment 1 and 2 were placed in plain tubes after collection, centrifuged at 2,500 X g at 4°C for 10 min. The supernatant was placed in cryovials, and stored at−20° until quantification of lidocaine and mepivacaine concentrations.

### Laboratory analyses

Concentration of lidocaine and mepivacaine was assessed using high-performance-liquid-chromatography with MS detection (HPLC MS). The method for quantification of LAs was based on a previously described method for LA quantification (19), which was modified for the present study's bioanalysis and Liquid chromatography-mass spectrometry (LC-MS). The chromatographic systems consisted of a Dionex ultimate 3,000 gradient pump, autosampler, column compartment and a diode array detector, coupled to a Finnigan TSQ Quantum Ultra triple quadrupole mass spectrometer (Thermo scientific, Hvidovre, Denmark). Methanol and formic acid (Sigma Aldrich, Soeborg, Denmark) and commercially available solutions of the same batch of lidocaine (AstraZeneca, Cambridge, UK), mepivacaine (AstraZeneca, Cambridge, UK) and ropivacaine (Fresenius Kabi, Bad Homburg, Germany) were used for method development. Ropivacaine was used as an internal standard (IS).

A Kinetex biphenyl column 100 Å 100.0 mm x 4.6 mm, PN 00D-4622-E0 (Phenomenex, Vaerloese, Denmark) was used for quantification of LAs. A binary gradient elution method and a flow of 0.25 mL/min was used. Mobile phase A consisted of 0.1% formic acid in deionized water (MilliQ, Sigma-Aldrich, St. Louis, Missouri). Mobile phase B consisted of 0.1% formic acid in MeOH. The gradient program was: 0–1 min. 10% mobile phase B, from 1 to 5 min. Mobile phase B was increased from 10 to 80% mobile phase B, from 5 to 7 min mobile phase B was kept constant at 80% mobile phase B. From 7 to 7.1 min, mobile phase B was decreased from 80 to 10%. From 7.1 to 11 min mobile phase B was kept constant at 10% (re-equilibration) yielding an analysis time of 11 min/sample. A volume of 5.0 μL was injected. A column temperature of 30°C, and an autosampler temperature of 15°C were used.

### Sample preparation

Stock solutions of mepivacaine and lidocaine (1 mg/mL) were prepared. A volume of stock solution was diluted with deionized water (milliQ, 18.2 MOhm/cm) as matrix free standards. An internal standard stock solution (100 ng/ml ropivacain in acetonitrile) was prepared. Standards in matrix were prepared using 10 μL of stock solution diluted with 90 μL SF. These were precipitated using 400 mL cold IS stock solution and centrifuged (Eppendorf centrifuge 5415R) for 10 min at 4°C, at 16,1 X g. Samples were prepared using 100 μL SF diluted with 400 mL IS stock solution and centrifuged as above. Blank samples were precipitated with 400 μL IS stock solution. Zero samples were precipitated with 400 μL acetonitrile. All samples and standards were diluted with a factor of 10 with milliQ water before injection (100 μL sample + 900 μL milliQ water).

### Validation of the LC-method

The validation of the method was performed largely following the ICH M10 guideline^1^. (accessed January 1, 2022). The following parameters were validated: Linearity repeatability and some inter-day reproducibility (A freshly prepared set of standards were prepared and run at start of run, halfway through, and, at end of run of each batch of samples analyzed). Linearity was checked by fitting first order or second order polynomial to the analytical response using commercially available software (Xcalibur^TM^ software version 2.2 SP1, Thermo Fischer, Waltham, Mass.). As the calibration curve proved to be slightly non-linear as was also evident from residuals plots, it was chosen to proceed with using a second order fit.

Further dilutions of some samples were performed in order to stay within the validated detection range (hence, dilution of samples was validated).

Stability of samples was evaluated by re-analyzing frozen samples after 2 months, which revealed no significant differences. All results were calculated with a 95 % confidence level, and no results fell outside the confidence level of the result of a previous/later run, corresponding to a probability of <0.05, that they are different. Likewise, no degradation of the analyte was observed in neither samples nor standards over a period of 2–3 days in the autosampler. In all samples, the concentration of the analyte differed < ± 15% from the nominal or previously determined values.

The limit of quantitation (LOQ) and limit of detection (LOD) was estimated from the lowest standards for both compounds and checked for each run to ensure that the quantitative analysis was performed above or at the quantitation limit which is not defined in the ICH M10 guideline, but can be inferred from the maximal allowed standard deviation of 20% for the lowest standard. We have chosen a more conservative value of 10% for the allowable standard deviation of the lowest standard, corresponding to the IUPAC definition of LOQ. All tested standards were above this limit (Supplementary Table 1).

### Calibration curve

A calibration curve was generated containing blank samples and zero samples (blank sample spiked with IS), at seven concentration levels of calibration standards using six replicates, from Lower limit of quantitation (LLOQ) to the Upper limit of quantitation (ULOQ). The LLOQ and ULOQ are not the absolute LOQs but the tested limits. The validated and tested range for both compounds were LLOQ = 0.5 μg/mL ULOQ = 15 μg/mL.

A second order polynomial (quadratic function) was used to model the calibration curve:


$$\begin{array}{l} Response=B2•C^{2}+B1•C+B0 \end{array}$$

Response is the ratio of the area corresponding to the analyte to the area corresponding to the IS. C is the concentration of the analyte. As shown from the second order coefficient (Supplementary Table 1), the calibration curve exhibits a slight downward curve, which is common with MS detection. Responses were weighted with 1/variance.

### Matrix effect

Three replicates of low and high quality control samples (4 levels), were tested on 3 consecutive days. The accuracy was within ±15% of the nominal concentration and the precision per cent coefficient of variation (%CV) was <15% in all cases.

### Statistical analysis

Chromatographic data analysis (Xcalibur^TM^ software version 2.2 SP1, Thermo Fischer, Waltham, Mass.) and calculation of calibration curves (and linearity check) (Prism version 9 for MacOS, Graphpad software, San Diego, California) was performed using commercially available statistical software programs (Xcalibur^TM^ software version 2.2 SP1, Thermo Fischer, Waltham, Mass. and Prism version 9 for MacOS, Graphpad software, San Diego, California). The statistical software was chosen because of the option to estimate confidence intervals for predicted (back calculated) concentrations.

Summary statistics (mean and standard deviation) were used for the presentation of concentrations of mepivacaine and lidocaine measured in SF of specific joints after intra-articular injection (Tables 1, 2) and calculated using GraphPad Prism. Similarly, data per joint and time point was tested for normality using Shapiro-Wilk test in GraphPad Prism.

**Table 1:** Mean (±SD) mepivacaine concentrations in equine joints after intra-articular injection of clinical doses of 2% mepivacaine.

**Joint**	**Injection dose [number of horses]**	**Mepivacaine (mg/mL)**	
		**Experiment 1**	**Experiment 2**
Middle carpal	10 ml [*n =* 5;7]	7.28 (1.00)	4.89 (0.81)
Tarsocrural	30 ml [*n =* 5]		6.02 (1.81)
	20 ml [*n =* 1]		4.56
Metacarpophalangeal	10 ml [*n =* 4]		3.02 (1.36)
	10 ml [*n =* 5]	7.45 (0.74)	
Distal interphalangeal, front	10 ml [*n =* 3]	10.55 (1.47)	6.95 (0.88)
	6 ml [*n =* 1]	5.01	
	5 ml [*n =* 1]		2.52
Distal interphalangeal, hind	5 ml [*n =* 4]	9.24 (1.18)	
	10 ml [*n =* 1]	13.38	

Numbers in parenthesis depicts ± standard deviation.

**Table 2:** Mean (±SD) lidocaine concentrations in equine joints after intra-articular injection of clinical doses of 2% lidocaine.

**Joint**	**Injection dose [number of horses]**	**Lidocaine (mg/mL)**	
		**Experiment 1**	**Experiment 2**
Middle carpal	10 ml [*n =* 5;7]	8.95 (1.94)	5.94 (1.33)
Tarsocrural	30 ml [*n =* 5]		7.89 (1.78)
	20 ml [*n =* 1]		3.33
Metacarpophalangeal	10 ml [*n =* 6]		4.66 (2.14)
	10 ml [*n =* 5]	10.19 (2.34)	
Distal interphalangeal, front	10 ml [*n =* 4]	17.00 (2.75)	8.19 (1.89)
	6 ml [*n =* 1]	11.38	
Distal interphalangeal, hind	5 ml [*n =* 4]	11.55 (3.17)	
	10 ml [*n =* 1]	17.80	

Numbers in parenthesis depicts ± standard deviation.

## Results

All data per joint and time point was normally distributed and given as range and/or mean ± SD. Likewise were time passed from joint injection to synovial fluid retrieval in both experiment 1 and 2 normally distributed and given as mean time point ± SD.

### Experiment 1

Synovial fluid was obtained 6.8 ± 1.8 min following intra-articular injection of mepivacaine and lidocaine. At this time point, concentrations of mepivacaine were 5.01–13.38 mg/mL (mean 8.18 ± SD 1.76 mg/mL) depending on joint and dose (Table 1). Concentrations of all lidocaine-injected joints were 6.46–19.62 mg/mL (mean 11.96 ± SD 3.89 mg/mL) (Table 2).

One DIP joint was excluded on the day of injection due to injection failure.

### Experiment 2

Synovial fluid was obtained 22.95 ± 4.1 min following intra-articular injection of mepivacaine and lidocaine. At this time point, concentrations of all mepivacaine-injected joints were 2.10–8.70 mg/mL (mean 4.97 ± SD 1.77 mg/mL) depending on joint and dose (Table 1) and concentrations of all lidocaine-injected joints were 2.94–10.40 mg/mL (mean 6.31± SD 2.23 mg/mL) (Table 2).

Concentrations of mepivacaine were 33, 61, and 34% lower for MC, MCP, and DIP joints, respectively, after 23 min in experiment 2 than after 6.8 min in experiment 1 (Table 1). For lidocaine, concentrations were 34, 48, and 52% lower in MC, MCP and DIP joint, respectively, after 23 min in experiment 2 than after 6.8 in experiment 1 (Table 2). Comparisons were made for those joints injected with 10 mL of local anesthetic.

## Discussion

The present study reveal *in vivo* concentrations of mepivacaine and lidocaine in a number of clinically relevant equine joints after single intra-articular injection as performed in equine orthopedic practice during lameness diagnosis. Previously, research within the field of equine lameness diagnosis with LA injections have revolved around specificity of intrasynovial analgesia (18, 20–23) and clinical effect of intrasynovial analgesia on solar pain (17, 24, 25) but not previously have LA concentrations in SF of injected joint cavities been assessed.

In spite of routine use of LAs in equine orthopedic practice, pharmacokinetic studies after intra-articular LA administration remain unexplored. Traditionally 5–10 mL of 2% mepivacaine/lidocaine solution have been used for intra-articular anesthesia of the DIP joint (1), 10 mL is recommended for the MCP joint (1), 5–10 mL for the MC joint (1) and 20–30 mL for the TC joint (1). In the present study, similar volumes have been used to mimic the clinical setting.

Our study reveals that SF concentrations of lidocaine and mepivacaine after routine intra-articular injections are above concentrations associated with cytotoxicity to articular cells but also sufficiently high to possess antimicrobial activities against common equine pathogens (11, 16).

Adverse effects of LAs on articular cartilage were first established after continuous intra-articular administration of bupivacaine in joints of humans (26) and have been recently established in horses after single intra-articular lidocaine and mepivacaine injection (12). A number of *in vitro* studies have confirmed toxicity of LAs to articular cells and sought to establish concentrations at which LAs exert their harmful effects (7–9, 11, 27–30). Toxic insults to equine chondrocytes and synoviocytes have been seen *in vitro* at concentrations as low as >5–10 mg/mL of mepivacaine and lidocaine (11). *In vitro*, investigations of cytotoxicity are carried out as two-fold dilutions, meaning that when *in vitro* results show cytotoxic effects after exposure to 10 mg/mL and not after exposure to 5 mg/mL, the exact concentrations at which a drug exhibits its cytotoxic effects lie somewhere in between the two investigated concentrations.

The results of the present study show that after a mean of 7 min the lidocaine concentrations of the majority of joints exceed those concentrations previously associated with toxic effects on fibroblast-like synoviocytes and chondrocytes *in vitro* (11). After a mean of 7 min, mepivacaine concentrations were either above concentrations previously associated with cytotoxicity (≥10 mg/ml) or within a two-fold dilution of that concentration (between 5 and 10 mg/ml), suggesting that not only mepivacaine administration of 10 mL to the DIP joint but also mepivacaine administration of 10 mL to the MC and MCP joint have the potential to result in toxic effects (Table 1). The fact that concentrations between the two-fold dilutions of 5–10 mg/mL affects articular cells negatively are supported by an *in vivo* study establishing a catabolic cartilage insult alongside joint inflammation after single-intra-articular injection of 10 mL of 2% lidocaine and mepivacaine to the equine MC joint (12).

After a mean of 23 min, administration of 30 mL mepivacaine and lidocaine to the TC joint, 10 mL to the DIP joint and 10 ml lidocaine to the MC joint resulted in SF LA concentrations above 5 mg/mL, potentially affecting cartilage negatively as previously described. In the remainder joints, concentrations were below 5 mg/mL, and therefore no longer considered to be toxic to articular cells as compared with *in vitro* results (11).

Another established side effect of LAs is the beneficial inherent antimicrobial activity (15, 31), which have also been established against equine clinical isolates (16). Lidocaine at the concentration of ≤5–10 mg/mL was previously effective against most (37/40) investigated equine clinical isolates (16). After a mean of 7 min, all measured concentrations of lidocaine in SF of the present study were > 5 mg/mL, proving clinically relevant concentrations in SF from an antimicrobial perspective. Mepivacaine at concentrations of ≤5–10 mg/mL had antimicrobial effect against several (27/40) tested equine isolates (16). In the present study, all SF concentrations were > 5 mg/mL after a mean of 7 min, suggesting a clinically relevant potential for an antimicrobial effect of mepivacaine in the joints. To minimize risk of iatrogen septic arthritis development, some clinicians include prophylactic antibiotics for routine joint injections, including those performed with LAs (32). The fact that LAs possess antimicrobial activities at the concentrations present in the SF should encourage those clinicians to avoid prophylactic antibiotics in routine LA joint injections.

Even though the present study is not a pharmacokinetic study, concentrations of mepivacaine and lidocaine were generally lower after a mean of 23 min than after a mean of 7 min. Concentrations of mepivacaine and lidocaine were 33–61 and 34–52%, respectively, lower after a mean of 23 min in experiment 2 compared with after a mean of 7 min in experiment 1. Although equine pharmacokinetics on LAs after intra-articular administration is unknown, these findings suggest that clearance from the joint cavity to the systemic circulation is fast. A rapid clearance of LAs from the joint cavity is supported by a canine study, which established a clearance of lidocaine from the elbow joint SF into serum to start already 5 min after intra-articular injection and further that serum concentrations peaked in 4/6 dogs 30 min after intra-articular injection (33).

Concentrations of lidocaine in SF after a mean of 7 min were generally higher than mepivacaine concentration (Tables 1, 2). The exact cause for this difference in concentration after administration is unknown; however, the physical and chemical properties of lidocaine and mepivacaine are most likely major contributors. Lidocaine is clinically known to have a faster onset of action and has a higher lipid solubility than mepivacaine, whereas mepivacaine has a higher protein binding capacity and a longer duration of action (34).

Concentrations at which LAs abolish lameness in different joints largely remain unknown. It has been estimated that mepivacaine concentrations of >0.3 mg/mL are sufficient to abolish equine joint related lameness (23). Accepting this estimate, and assuming a similar number/concentration is applicable for lidocaine, the concentrations revealed in the present study for mepivacaine and lidocaine, respectively, is 17–35 and 30–57 times that necessary to abolish lameness in the horse at a mean of 7 min after intra-articular injection. At a mean of 23 min after intra-articular injection, concentrations of mepivacaine and lidocaine were 10–23 and 11–27 times that necessary to abolish lameness (Tables 1, 2). Clinicians need to be able to rely on the efficacy of the performed intra-articular block. Therefore, doses are often higher than necessary to ensure joint concentrations to remain high enough to abolish lameness for a period of time, during which lameness diagnostics are ongoing. Considering that clearance of LAs from joint cavities is fast (33), administration of larger doses probably is sensible to ensure that the concentration within the joint cavity remains above the effective dose while lameness examinations proceed. Nonetheless, since results of our study show that concentration in SF remains ≥ 10 times higher than that necessary to abolish lameness at a mean of 23 min after intra-articular administration, this may suggest that lowering the doses could be an option to reduce toxic side effects without compromising the anesthetic effect of LAs essential for lameness diagnosis. Establishing minimum effective doses of LAs for lameness abolishment of different joints should be further investigated alongside pharmacokinetics after intra-articular LA injections for optimal clinical recommendations. While lowering of doses for intra-articular anesthesia likely would result in reduced intra-articular toxicity, a simultaneous decrease in the positive antimicrobial activity is expected as both observed side effects are concentration dependent. However, concerning side effects, reducing adverse effects while maintaining anesthetic effect should remain the principal purpose during lameness diagnosis, and septic arthritis prophylaxis should be based on proper preparation and technique.

Our study had more limitations, some of which are common for many *in vivo* studies. Firstly and mainly, we included horses both under general anesthesia (experiment 1) and standing sedation (experiment 2) to maximize the information obtained per experimental animal (those under general anesthesia provided information for two studies as they were also part of another study). Hereby we reduced the overall number of experimental animals. Although in the clinic, lameness diagnosis is performed in the standing horse, and not under general anesthesia, we do not consider this a limitation to our study, as our study aim was to investigate concentrations in synovial fluid after injection at two different time points. Including horses under general anesthesia gave us the opportunity to inject (and retrieve SF from) multiple joints quickly compared with in the standing horse. To mimic *in vivo* conditions and to stimulate distribution of LAs in the SF of the joint cavity, we flexed and extended joints of horses under general anesthesia thoroughly 30 times after injection of LA prior to aspiration from the joint cavity. The main difference between groups (general anesthesia vs. standing sedation) was the time from injection of LAs to aspiration, which was much quicker for horses under general anesthesia than during standing sedation. Secondly, the number of joints included in the study for specific volumes were between one and seven. Ideally, we would have wanted multiple joints represented for each included volume but as more horses and joints were not available for the study we chose to include most horses/joints for doses used most often clinically (for instance 10 ml for the MC and MCP joint) (1). Nonetheless, as recommendations for some joints are made as intervals as described previously, we wanted to include a subset of different volumes whenever possible to maximize output of information from each individual research animal. The reader should bear in mind that some of the investigated joint volumes were performed in only one joint, which is a limitation to the study. Lastly, we injected multiple joints of horses with LAs at the same time, and therefore cannot exclude that this may have affected results. Nonetheless, based on a previous study performed in the dog, which revealed that maximum lidocaine concentration in serum after intra-articular injection of 15 ml 2% lidocaine was 1 μg/ml (33), we consider this potential carryover effect of clinically relevant LA concentration from one joint to another clinically insignificant.

In conclusion, clinically used intra-articular doses of lidocaine and mepivacaine in several equine joints result in concentrations higher than those previously reported to be toxic to articular cells. Synovial fluid LA concentrations after injection also exceed concentrations previously associated with beneficial antimicrobial activities rendering use of simultaneous prophylactic antibiotics unnecessary. Until single-dose LA pharmacokinetics and minimum effective anesthetic dose in joints are elucidated, clinicians are encouraged to use doses in the lower range of recommended doses to avoid adverse cytotoxic effects.

## Data availability statement

The raw data supporting the conclusions of this article will be made available by the authors, without undue reservation.

## Ethics statement

The animal study was reviewed and approved by the Ethical Committee of the University of Copenhagen Large Animal Teaching Hospital and the Danish Animal Experiments Inspectorate (License No. 2015-15-0201-00608).

## Author contributions

DA and EJ conducted the clinical study. CC conducted the analytical part. All authors contributed to and accepted this submission.

## Conflict of interest

The authors declare that the research was conducted in the absence of any commercial or financial relationships that could be construed as a potential conflict of interest.

## Publisher's note

All claims expressed in this article are solely those of the authors and do not necessarily represent those of their affiliated organizations, or those of the publisher, the editors and the reviewers. Any product that may be evaluated in this article, or claim that may be made by its manufacturer, is not guaranteed or endorsed by the publisher.

## Footnotes

^1^ICH M10 guideline: https://www.ema.europa.eu/en/ich-m10-bioanalytical-method-validation#current-version-section.
