# Supplementary material for: The concentration of lidocaine and mepivacaine measured in synovial fluid of different joints of horses after single intra-articular injection
Source: Front Vet Sci. 2022 Nov 10;9:1007399. doi: 10.3389/fvets.2022.1007399 (PMC9684627; doi:10.3389/fvets.2022.1007399)
Supplement: Supplementary file 1 [file Table_1.docx]

**Supplementary file**

**Table 1 Summary of validation of calibration results**

**a. Mepivacaine**

| Function  parameters | Value | Lower 95 % confidence limit | Lower 95 % confidence limit |  |
| --- | --- | --- | --- | --- |
| B0 | 0.09070 | -0.02903 | 0.2104 |  |
| B1 | 1.107 | 1.073 | 1.140 |  |
| B2 | 0.01277 | -0.01443 | -0.01111 |  |
| Degrees of freedom | 39 |  |  |  |
| R^2^ | 0.9990 |  |  |  |
| RMSE | 0.1940 |  |  |  |
| Kolmogorov-Smirnov (distance) | 0.1135 |  |  |  |
| P value | >0.1000 |  |  |  |
| Passed normality test (alpha=0.05)? | Yes |  |  |  |
|  | C (mg/mL) | Accuracy (%) | CV (%) | Compliance with ICH M10 |
| Std1 | 0.500 | 10.3 | 6.2 | Yes, within ±20 % |
| Std2 | 1.000 | 6.6 | 4.2 | Yes, within ±15 % |
| Std3 | 2.500 | -8.5 | 6.4 | Yes, within ±15 % |
| Std4 | 7.0 | 0.2 | 2.3 | Yes, within ±15 % |
| Std5 | 10.000 | 2.7 | 2.6 | Yes, within ±15 % |
| Std6 | 15.000 | -2.1 | 3.3 | Yes, within ±15 % |
| Std7 | 20.000 | 0.6 | 7.0 | Yes, within ±15 % |
| Estimater LOD (mg/mL) | 0.02 |  |  |  |
| Estimated LOQ (mg/mL) | 0.07 |  |  |  |

**b. Lidocaine**

| Function  parameters | Value | Lower 95 % confidence limit | Lower 95 % confidence limit | |  |
| --- | --- | --- | --- | --- | --- |
| B0 | 0.02676 | -0.1128 | 0.1663 | |  |
| B1 (mL/ mg) | 0.671 | 0.632 | 0.798 | |  |
| B2 (mL^2^/ mg^2^) | -0.007202 | -0.009136 | -0.005268 | |  |
| Degrees of freedom | 39 |  |  |  |  |
| R^2^ | 0.9963 |  |  |  |  |
| RMSE | 0.2261 |  |  |  |  |
| Kolmogorov-Smirnov (distance) | 0.2476 |  |  |  |  |
| P value | >0.1000 |  |  |  |  |
| Passed normality test (alpha=0.05)? | Yes |  |  |  |  |
|  | C (mg/mL) | Accuracy (%) | CV (%) | Compliance with ICH M10 | |
| Std1 | 0.500 | 10.3 | 8.8 | Yes, within ±20 % | |
| Std2 | 1.000 | 6.6 | 4.2 | Yes, within ±15 % | |
| Std3 | 2.500 | -8.5 | 4.9 | Yes, within ±15 % | |
| Std4 | 7.0 | 0.2 | 5.7 | Yes, within ±15 % | |
| Std5 | 10.000 | 2.7 | 2.7 | Yes, within ±15 % | |
| Std6 | 15.000 | -2.1 | 3.4 | Yes, within ±15 % | |
| Estimater LOD (mg/mL) | 0.035 |  |  |  |  |
| Estimated LOQ (mg/mL) | 0.09 |  |  |  |  |
